# Biofeedback Physical Therapy With the Hybrid Assistive Limb (HAL) Lumbar Type for Chronic Low Back Pain: A Pilot Study

**DOI:** 10.7759/cureus.23475

**Published:** 2022-03-25

**Authors:** Yoshihiro Yasunaga, Ren Koizumi, Taro Toyoda, Masao Koda, Naotaka Mamizuka, Yoshiyuki Sankai, Masashi Yamazaki, Kousei Miura

**Affiliations:** 1 Department of Orthopaedic Surgery, Faculty of Medicine, University of Tsukuba, Tsukuba, JPN; 2 Department of Rehabilitation Medicine, Baseball & Sports Clinic, Kawasaki, JPN; 3 Department of Orthopaedics, Baseball & Sports Clinic, Kawasaki, JPN; 4 Center for Cybernics Research, University of Tsukuba, Tsukuba, JPN

**Keywords:** exoskeleton robots, hip flexibility, biofeedback physical therapy, hybrid assistive limb, low back pain

## Abstract

Objective

There are many treatments for chronic low back pain, including various medications, exercise therapy, orthotics, and surgery, but no treatment is definitive. We hypothesized that biofeedback therapy using the hybrid assistive limb (HAL) lumbar type would have some immediate effects on chronic low back pain. The purpose of this pilot study was to assess whether immediate changes in low back pain and hip flexibility and any other adverse events would occur following the HAL biofeedback physical therapy.

Methods

This was a single-center, pilot, prospective, single-arm study of outpatient biofeedback physical therapy using the HAL lumbar type for patients with chronic low back pain. Patients underwent a 10-minute biofeedback physical therapy (lumbar flexion-extension, sit-to-stand, and squat) with the HAL lumbar type (in one session). The visual analog scale (VAS) score of low back pain during lumbar flexion, extension, lateral bending, and rotation was evaluated. The finger-to-floor distance (FFD), straight leg raising test (SLR), and the Thomas test were measured to assess hip flexibility.

Results

All 35 participants (14 men and 21 women) (100%) conducted a biofeedback HAL therapy session using the HAL lumbar type. No participant had deterioration of low back pain. No adverse events occurred. After the biofeedback therapy using the HAL lumbar type, SLR demonstrated a significant positive change with large effect size and sufficient power. Lumbar VAS during lumbar flexion and extension and FFD showed a significant positive change with medium effect size and adequate power.

Conclusions

Biofeedback therapy using the HAL lumbar type is an option for intervention in chronic low back pain.

## Introduction

Most people have experienced low back pain. Low back pain lasting more than three months is considered chronic pain [1]. Low back pain is one of the most common musculoskeletal disorders among persons with chronic pain. Many treatments have been used for chronic low back pain, including various medications, exercise therapy, orthotics, and surgery, but no treatment is definitive. It is essential to find an effective way to prevent and treat low back pain to reduce the burden on patients and national healthcare expenditures.

Many exoskeleton robots have been developed. The hybrid assistive limb (HAL) (Cyberdyne, Ibaraki, Japan) is a unique wearable exoskeleton robot controlled by the wearer’s intent to move. The product’s noninvasive sensors can detect very faint bioelectrical signals (BES) that reflect the wearer’s intention on the surface of their skin. Even if patients have difficulty moving, this unique control system allows them to control their desired movements with their voluntary commands [2]. There are three types of HAL systems: the HAL lower limb type, the HAL single-joint type, and the HAL lumbar type. The HAL has been reported to improve function even in the chronic stage of disorders for spinal cord injury [3], stroke [4,5], cerebral palsy [6,7], and progressive neuromuscular disease [8] using the HAL lower limb type. Patients who undergo HAL therapy after knee surgery have been reported as without pain and felt pain relief after HAL therapy using the HAL single-joint type [9,10] and the HAL lower limb type [11,12]. The HAL is designed based on the “interactive biofeedback” (iBF) hypothesis to facilitate movements [13].

The present research focuses on the HAL lumbar type. The HAL lumbar type was initially developed to support and reduce the burden of heavy patient loads for caregivers [14]. The HAL lumbar type can reduce the load on the lower back by supporting the hip flexion and extension movements. To date, the possibility of reducing subjective lumbar fatigue and improving lifting performance in repetitive lifting movements [15], repetitive snow shoveling movements [16], and simulated patient transfer movements [17] has been shown in healthy adults by using the HAL lumbar type. Furthermore, the use of the HAL lumbar type by healthy volunteers may reduce cardiopulmonary strain during a stand-up exercise [18]. The HAL lumbar type has not only been used to decrease the burden of a heavy load but has also been used in biofeedback therapy for physically frail patients [19] and those with locomotive syndrome [20]. Biofeedback therapy is based on electromyographic biofeedback, which detects muscular electrical activity through BES and feeds back the magnitude of the voluntary muscle activity. Considering previous studies, we hypothesized that biofeedback therapy using the HAL lumbar type would have some immediate effects on chronic low back pain. However, to our knowledge, there is no report of using the HAL lumbar type for chronic low back pain. The purpose of this pilot study was to assess the immediate effect on low back pain and hip flexibility and adverse events following exercise therapy with the HAL lumbar type.

## Materials and methods

Participants

**Figure 1:**
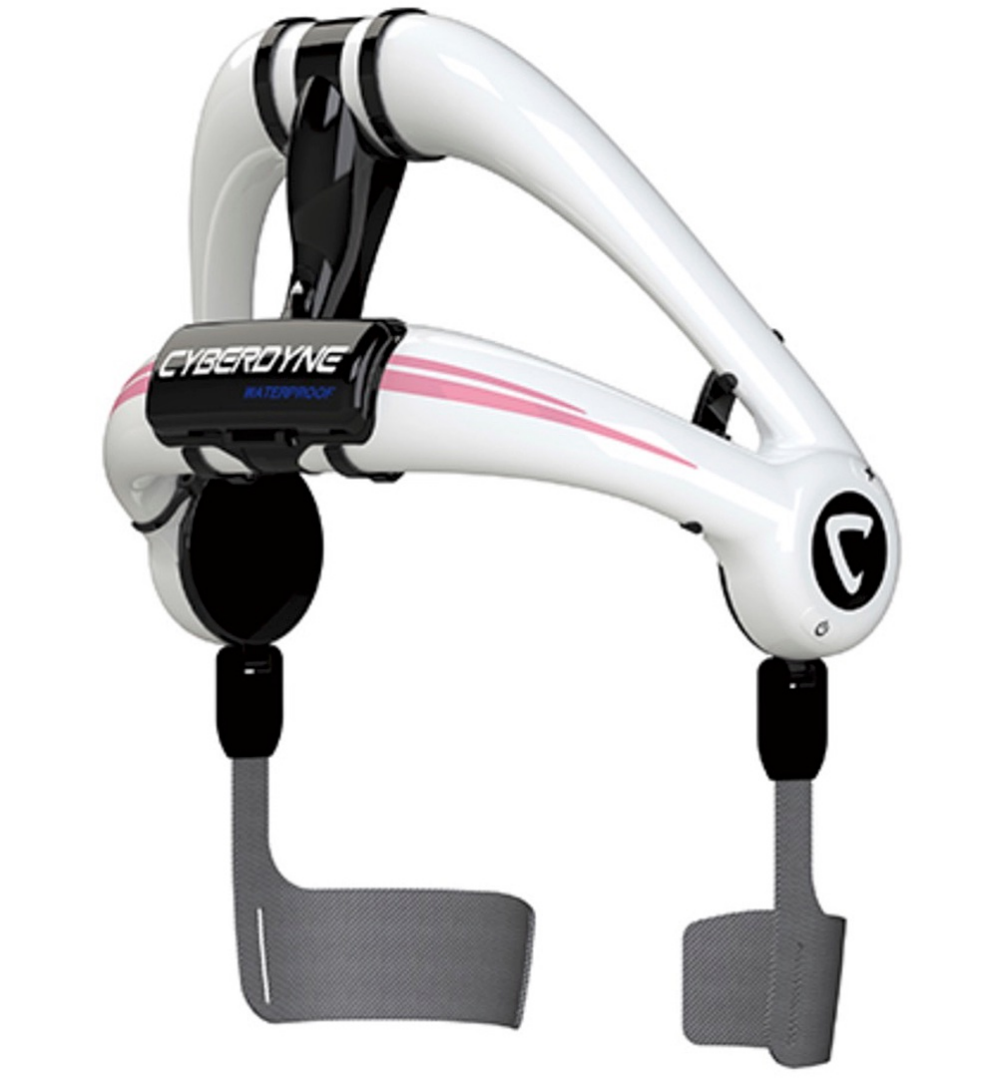
The HAL lumbar type

This was a single-center, pilot, prospective, single-arm study of outpatient biofeedback physical therapy using the HAL lumbar type for patients with chronic low back pain. The following inclusion criteria were used: (1) aged 18 years or older and (2) complaining of low back pain over three months. The exclusion criteria were as follows: (1) ineligible body size for the HAL lumbar type (e.g., waist circumference > 120 cm and thigh circumference > 80 cm), (2) dermopathy at the site where the electrodes of the HAL lumbar type are applied, (3) cardiopulmonary failure that interferes with exercise therapy, and (4) severe lower limb disorders affecting exercise therapy. We obtained informed consent from all participants. This study was approved by the Institutional Review Board of the Cyberdyne institute (IRB approval number 210003) and was conducted according to the principles of the Declaration of Helsinki. The HAL lumbar type is shown in Figure 1.

The HAL lumbar type consists of an exoskeleton, a power unit, and lumbar and thigh molds. The actuators of the power unit are placed at the bilateral femoral greater trochanters of the wearer and generate torque to assist the extension of the hip joint. In addition, a triaxial accelerometer built into the exoskeleton frame can detect the absolute angle of the trunk, while the angle sensor and potentiometer built into the power unit can detect the relative angle of the hip joint. With these mechanisms, the HAL lumbar type can assist the wearer’s movements according to the level and timing of the torque. Furthermore, the HAL lumbar type is equipped with two hybrid control systems: a cybernic voluntary control (CVC) and a cybernic autonomous system (CAC). The CVC system can control the actuator torque of the HAL to augment the joint torque of the wearer according to voluntary muscle activity by detecting the wearer’s motion through a BES. Alternatively, the CAC system can support and reduce the moment caused by trunk flexion onto the lumbar back. Hara et al. reported these two control systems in detail [14]. An advantage of the HAL lumbar type is its lightness. Its external dimensions are 292 mm (length) × 450 mm (width) × 522 mm (height), and it weighs only 3.1 kg, including batteries. The hip joint range of motion is 30° of extension and 130° of flexion. The power source is the original battery, running for about 4.5 hours on a two-hour charge. In addition, the HAL lumbar type has a simple structure and can be installed by the wearer alone in about three minutes.

Biofeedback physical therapy with the HAL lumbar type

**Figure 2:**
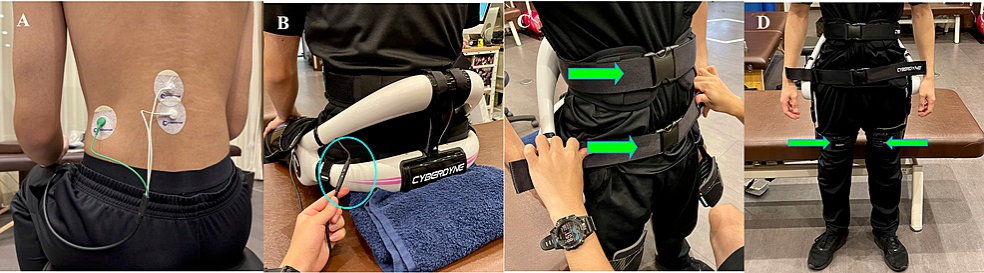
How to wear the HAL lumbar type (A) Attach the electrodes on the skin over the lumbar erector spinae muscle to detect muscle action potentials. (B) Connect the electrode cords to the body of the HAL lumbar type. (C) Fix the lumbar mold at the front of the abdomen. (D) Fix the thigh mold on both thighs.

Biofeedback therapy using the HAL lumbar type comprised lumbar flexion and extension exercise in a sitting position, sit-to-stand exercise, and squat exercise. First, electrodes were attached to the lumbar erector spinae muscle of the participants (Figure 2).

**Figure 3:**
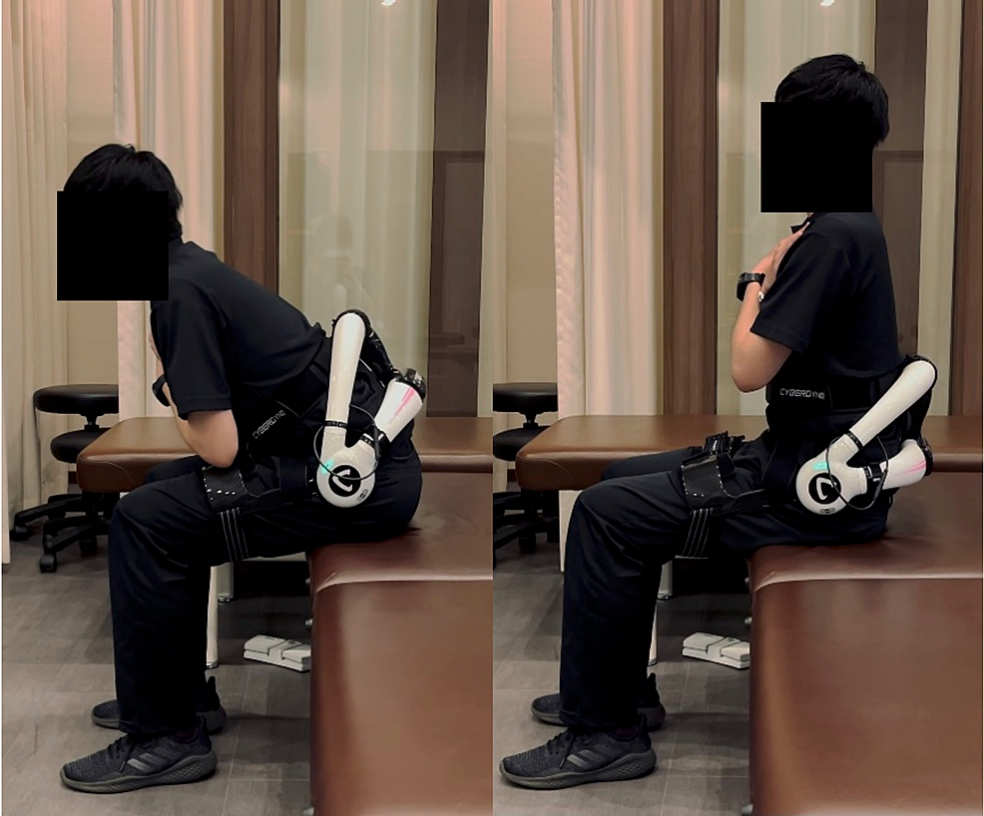
Repetitive lumbar flexion and extension exercise with the HAL lumbar type

Lumbar flexion and extension exercises were repeated for 90 seconds in a sitting position (Figure 3).

**Figure 4:**
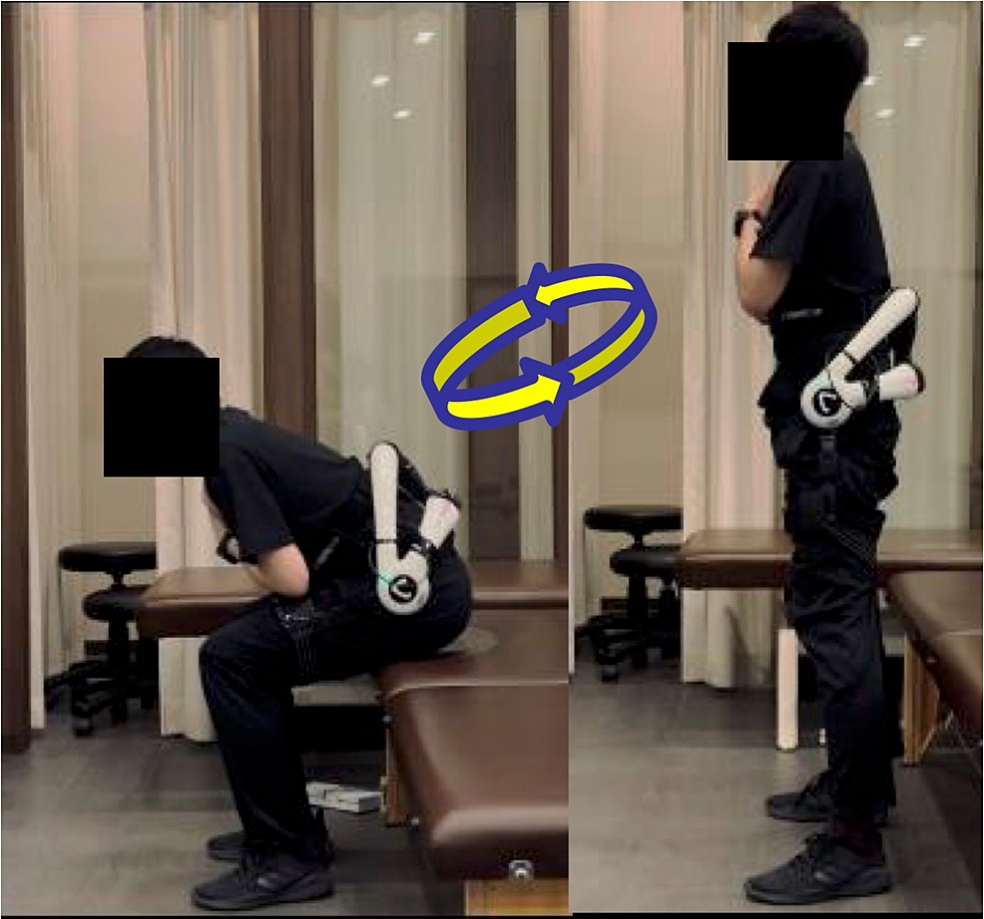
Repetitive sit-to-stand exercise with the HAL lumbar type

Subsequently, repetitive movements from sitting to standing were performed for 90 seconds (Figure 4).

**Figure 5:**
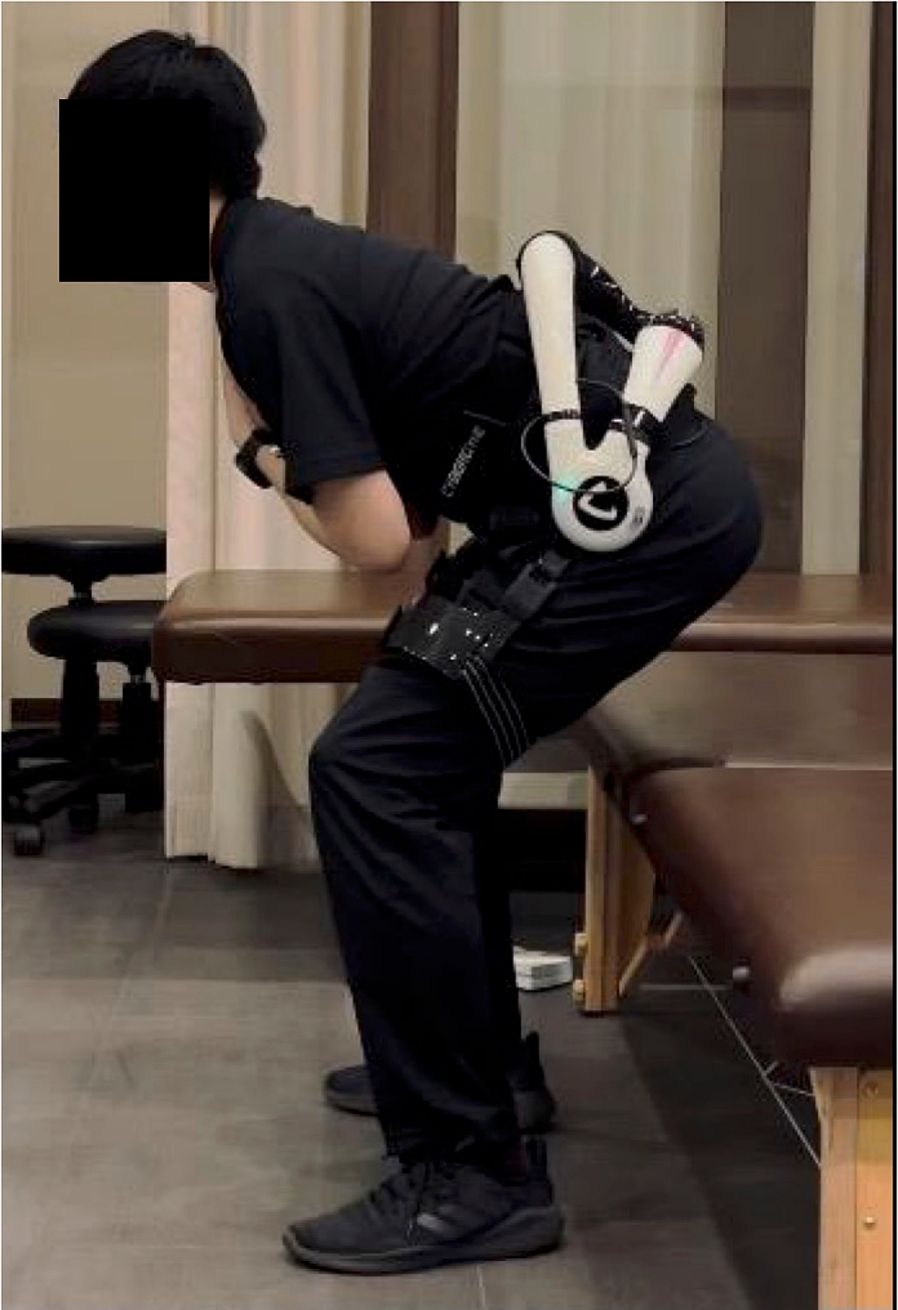
Continuous squat with the HAL lumbar type

Finally, a series of three sets of 10 squats on the ground was performed (Figure 5).

All types of exercise were performed at a pace comfortable for the subject using the HAL lumbar type. The total duration of the biofeedback HAL therapy was about seven minutes, including rest time.

Outcome measures

We assessed low back pain and hip flexibility immediately before and after HAL treatment (pre-HAL and post-HAL). A visual analog scale (VAS) score for low back pain during lumbar flexion, extension, lateral bending, and rotation was evaluated. The finger-to-floor distance (FFD), straight leg raising test (SLR), and Thomas test were measured to evaluate hip flexibility. The participants were assessed for low back pain and hip flexibility without HAL.

Statistical analysis

Low back pain and hip flexibility before and after HAL were compared using the Wilcoxon signed-rank test pre- and post-HAL use. All statistical analyses were performed using the JMP software package version 14.0.0 (SAS Institute, Cary, NC, USA). All tested differences were considered significant at p < 0.05.

## Results

In the present study, we recruited 35 participants (14 men and 21 women) with chronic low back pain. Their mean age ± standard deviation (SD) was 58 ± 16 years (range: 20-83 years), their mean height ± SD was 161.8 ± 8.7 cm (range: 142-180 cm), and their weight ± SD was 61.9 ± 11.9 kg (range: 41-98 kg). The clinical diagnoses were lumbar canal stenosis in 10 cases, disc herniation in seven, intervertebral disc disease in seven, nonspecific low back pain in five, lumbar spondylosis in three, lumbar spondylolisthesis in two, and lumbar spondylolysis in one.

All 35 participants completed biofeedback therapy using the HAL lumbar type. No participant had increased low back pain. No other adverse events occurred, including external injuries, falling, skin disorders, uncontrollable cardiovascular or respiratory disorders, or other health disorders directly related to the biofeedback therapy.

The statistical data of low back pain and hip flexibility are summarized in Table 1.

**Table 1:** Statistical data of low back pain and hip flexibility (n = 35) Values are mean ± standard deviation. VAS: visual analog scale, FFD: finger-to-floor distance, SLR: straight leg raising test. *p < 0.05, **p < 0.01

Outcome measures		Pre-HAL	Post-HAL	Effect size	Power
Lumbar VAS (cm)					
	Flexion	1.14 ± 1.49	0.41 ± 0.81**	0.57	0.89
	Extension	1.65 ± 1.98	0.71 ± 1.17**	0.55	0.86
	Right lateral bending	1.38 ± 2.17	0.61 ± 1.34**	0.41	0.62
	Left lateral bending	1.77 ± 2.48	0.77 ± 1.52**	0.42	0.74
	Right rotation	0.63 ± 1.68	0.22 ± 0.76**	0.28	0.35
	Left rotation	0.77 ± 1.68	0.23 ± 0.71**	0.37	0.55
FFD (cm)		7.6 ± 13	0.30 ± 13.6**	0.57	0.90
SLR (°)					
	Right	76 ± 13	91 ± 14**	1.05	0.99
	Left	76 ± 13	89 ± 13**	0.99	0.99
Thomas test (cm)					
	Right	2.9 ± 2.6	2.2 ± 2.3**	0.28	0.36
	Left	3.2 ± 2.7	2.7 ± 2.4**	0.31	0.42

After this biofeedback therapy using the HAL lumbar type, all outcome measures were improved significantly. SLR demonstrated a significant positive change with large effect size and sufficient power. Lumbar VAS during lumbar flexion and extension and FFD showed a significant positive change with medium effect size and sufficient power.

## Discussion

In the present pilot study, 35 participants with chronic low back pain underwent biofeedback therapy using the HAL lumbar type, including lumbar flexion and extension exercise in a sitting position, sit-to-stand exercise, and squat exercise. All participants could complete all exercises without adverse events such as increased low back pain. Furthermore, hip flexibility and low back pain during lumbar flexion and extension improved significantly immediately after the HAL treatment.

It has been recognized as challenging to choose an effective treatment for back pain [21]. The treatment for chronic low back pain remains controversial. We focused on hip-specific exercise therapy for patients with chronic low back pain. It has been reported that patients with chronic nonspecific low back pain have a small range of motion in hip extension [22]. Moreover, patients with chronic low back pain have been reported to have greater hip tightness than healthy controls [23]. These reports suggest that reduced hip flexibility may be associated with chronic low back pain. Lee and Kim found that patients with chronic low back pain who undertook active resistance exercises of hip joints using elastic bands had improved low back pain and Oswestry Disability Index (ODI) scores [24]. Bade et al. described a randomized controlled trial in patients with low back pain for which a combination of lumbar and hip exercises significantly reduced low back pain compared with exercise treatment for the lumbar spine alone [25]. Therefore, hip exercise may effectively reduce chronic low back pain.

In the present study, a single exercise session by patients with chronic low back pain using the HAL lumbar type showed immediate improvement in hip flexibility and low back pain during lumbar movement. There have been several reports on exercise treatment using the HAL lumbar type. Kotani et al. conducted a study of biofeedback core exercises using the HAL lumbar type in frail patients with Parkinson’s disease [19]. The patients wearing the HAL lumbar type performed five sessions of core and squat exercises and showed significant improvement in motor function, including a timed up and go test (TUG). Miura et al. also reported exercise therapy using the HAL lumbar type for participants with locomotive syndrome [20]. The participants performed 12 sessions of three kinds of exercises (sit-to-stand, lumbar flexion-extension, and gait training), which improved their balance function. However, to our knowledge, there have been no reports of exercise therapy using the HAL lumbar type for patients with chronic low back pain. Heavy work with the HAL lumbar type for healthy subjects has shown a reduction in lumbar load [15-17,26]. Watanabe et al. also reported that the Borg scale, a subjective measure of exercise intensity, decreased when the HAL lumbar type was used in squatting exercises [18]. These reports suggest that exercise therapy using the lumbar HAL can reduce lumbar load and exercise intensity and may be effective for patients with chronic low back pain. In the present study, a single session of biofeedback physical therapy using the HAL lumbar type showed immediate improvement in SLR and FFD with a medium to large effect size. The improvement in hip flexibility might have led to decreased back pain in flexion and extension.

**Figure 6:**
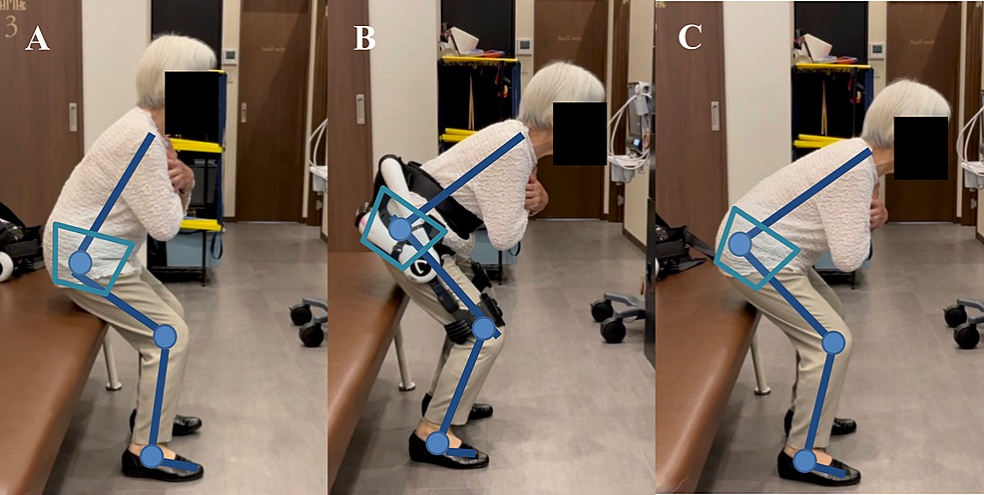
Representative patient squatting with the HAL lumbar type (A) Pre-HAL therapy (B) During the biofeedback physical therapy using the HAL lumbar type (C) Post-HAL therapy

In a representative case, the patient’s hip flexion angle widened in the anterior pelvic tilt posture during squatting with the HAL lumbar type (Figure 6A, 6B). The patient’s hip flexion angle remained widened in the anterior pelvic tilt posture during squatting even after removing the HAL lumbar type (Figure 6C).

**Figure 7:**
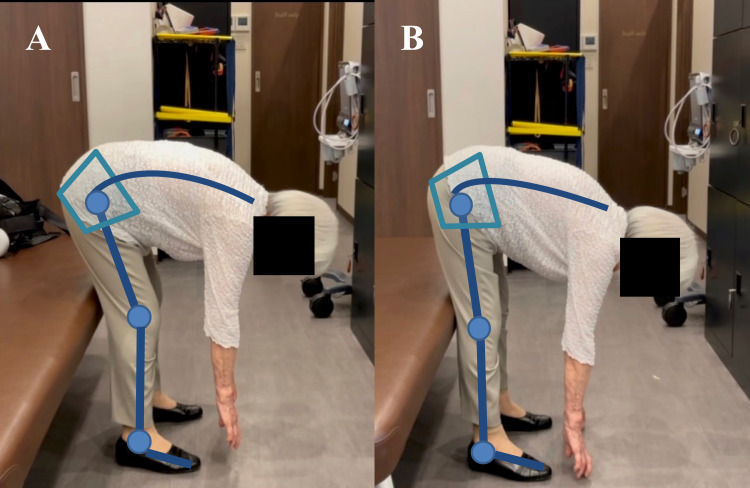
Representative patient in voluntary lumbar flexion movements (A) Pre-HAL therapy (B) Post-HAL therapy

This change in posture during squatting is thought to be due to the biofeedback of the HAL lumbar type, which could promote hip joint motion. In addition, this patient was in a posture of posterior pelvic tilt with knee joint flexion during lumbar flexion voluntary movement before the HAL exercise (Figure 7A). The patient changed their posture of anterior pelvic tilt with knee joint extension during voluntary lumbar flexion movement after the HAL exercise (Figure 7B).

This change indicates that hamstring tightness was improved after biofeedback physical therapy using the HAL lumbar type.

We hypothesized that the biofeedback mechanism of the HAL lumbar type would stimulate appropriate intrinsic receptors and suppress abnormal spinal reflexes through repeated feedback of movements without pain. This result is considered due to the characteristics of HAL, which can reduce the wearer’s motion by detecting the nerve and muscle action potentials of the lumbar erector spinae muscles while adjusting the strength and timing of the torque. The reduction of coordinated joint motion with no pain using the HAL lumbar type in accordance with the wearer’s intention is thought to induce positive biofeedback to the wearer’s nerves and muscles called the IBF hypothesis to facilitate movements [13]. This novel mechanism of the HAL lumbar type is expected to provide optimal biofeedback therapy for chronic low back pain.

The present study is limited because we did not evaluate the carryover effect, pelvic tilt angle, or range of motion of hip flexion using objective measures such as motion analysis, so we could not identify the clinical impact and cause of improvement. We had no available follow-up data. These data are important for evaluating the long-term outcome. This study did not recruit patients exercising without the HAL lumbar type as controls, and we could not measure the efficacy of the biofeedback therapy using the HAL lumbar type. Future investigation of these issues with a larger sample size is necessary to confirm our findings.

## Conclusions

All 35 participants with chronic low back pain conducted biofeedback therapy using the HAL lumbar type without deterioration of low back pain or any other adverse events. Immediately after a single session of the HAL exercise, SLR demonstrated a significant positive change with a large effect size and sufficient power. Lumbar VAS during lumbar flexion and extension and FFD also showed a significant positive change with medium effect size and sufficient power.

These findings suggest that biofeedback therapy using the HAL lumbar type might be an option for intervention in patients with chronic low back pain. Further studies are needed to elucidate the effect of biofeedback therapy using the HAL lumbar type for chronic low back pain.
